# Identification of protein-coding genes associated with metastatic prostate cancer

**DOI:** 10.1530/ERC-25-0070

**Published:** 2025-06-26

**Authors:** Mina Sattari, Hanna Rauhala, Leena Latonen, William B Isaacs, Matti Nykter, G Steven Bova, Juha Kesseli, Tapio Visakorpi

**Affiliations:** ^1^ Faculty of Medicine and Health Technology, Tampere University and Tays Cancer Center, Tampere University Hospital, Tampere, Finland; ^2^ Institute of Biomedicine, University of Eastern Finland, Kuopio, Finland; ^3^ Foundation for the Finnish Cancer Institute, Helsinki, Finland; ^4^ The James Buchanan Brady Urological Institute, Johns Hopkins School of Medicine, Baltimore, Maryland, USA; ^5^ Fimlab Laboratories Ltd, Tampere University Hospital, Tampere, Finland

**Keywords:** protein-coding gene, metastatic prostate cancer

## Abstract

Prostate cancer (PCa) is a significant cause of male mortality worldwide. Since metastases are the underlying cause of lethality, identifying markers for metastatic potential would be highly valuable. To address this issue, we set out to identify protein-coding genes with metastasis-specific expression changes in PCa. We employed a previously reported unique cohort consisting of metastases from castration-resistant prostate cancer (mCRPC) and matching primary tumors. Our comprehensive gene expression analyses identified 85 differentially expressed genes (DEGs) associated specifically with mCRPC, comprising 63 upregulated and 22 downregulated genes. Investigation of the transcription factors (TFs), such as the androgen receptor and its co-regulators FOXA1 and HOXB13, known to be important in prostate tumorigenesis, revealed their involvement in the differential expression of these genes. Furthermore, we identified enriched binding sites for nine TFs, namely EZH2, SUZ12, TLE3, TP63, CBX7, RNF2, SP140, JARID2, and CBX8, in the regulatory regions of the DEGs. Analysis of progression-free survival of prostatectomy-treated men highlighted 16 DEGs with significant prognostic value. Of these, three genes (FRMPD1, TMEM18, and ZNHIT3) were independent prognostic markers of biochemical recurrence. TMEM18 has putative androgen receptor-binding sites in its promoter region, and analysis of LNCaP cells following stimulation with dihydrotestosterone revealed a significant upregulation of TMEM18, confirming the androgen regulation of the gene. Furthermore, we confirmed the prognostic significance of TMEM18 expression at the protein level with immunohistochemistry (IHC) in a primary PCa tumor cohort. In conclusion, we identified 85 mCRPC-associated genes and showed that TMEM18 has prognostic value in early PCa.

## Introduction

Prostate cancer (PCa) is the second most common cause of male cancer-related death worldwide (Jemal *et al.* 2008). PCa is known for its significant genetic heterogeneity, characterized by numerous genomic changes (Berger *et al.* 2011, Mateo *et al.* 2020). Clinicians face the challenge of classifying tumors, which vary from indolent to extremely invasive, when dealing with patients diagnosed with PCa. Distinguishing between indolent and aggressive tumors with the potential to metastasize is essential for deciding on treatment (Eickelschulte *et al.* 2022).

Several biomarkers, such as prostate-specific antigen (PSA), have been discovered for diagnostic purposes (Hernández & Thompson 2004). Serum PSA is increased in PCa and also in benign prostatic hyperplasia (BPH), and it has limited value in distinguishing aggressive and non-aggressive cases (Etzioni *et al.* 2002, Finne *et al.* 2010, Phan *et al.* 2020). This highlights the need to identify alternative biomarkers for PCa.

The progress of genomic technology has greatly enhanced our understanding of tumor biology, prompting the search for more precise cancer biomarkers. The extensive documentation of differences in gene and protein expression between normal and malignant prostate tissues has provided a useful source of potential biomarkers for diagnostic, prognostic, and risk stratification purposes (Dhanasekaran *et al.* 2001, Cazares *et al.* 2002, Ernst *et al.* 2002, Wei *et al.* 2022).

In the present study, we aimed first to identify protein-coding genes associated with castration-resistant prostate cancer (mCRPC) metastases. We identified a distinct set of differentially expressed genes (DEGs) that show significant differences in expression between metastases and primary tumors. We investigated the regulation of the DEGs using multiple datasets. Next, we assessed the prognostic significance of the metastasis-associated genes in a prostatectomy-treated cohort, first at the mRNA level and subsequently at the protein level.

## Materials and methods

### mCRPC samples, prostatectomy and cell line samples, and sequencing

For this study, a previously published mCRPC cohort consisting of 105 samples obtained from autopsies of 25 PCa patients with metastatic castration-resistant prostate cancer (mCRPC) was utilized (Sattari *et al.* 2023). Of the 25 patients, primary tumor samples were available for six: five patients had one primary tumor sample each, and one patient had two. The metastases originated from different sites: liver (*n* = 12), adrenal gland (*n* = 7), bone (*n* = 18), subdural (*n* = 6), and lymph node (*n* = 39). Normal samples from the liver (*n* = 14) and adrenal gland (*n* = 2) from 15 patients in the cohort were also included (EGAS00001006959) (Supplementary Table S1 (see section on Supplementary materials given at the end of the article)). In addition, RNA-seq data from the publicly available dataset GSE72815 were utilized for 14 normal bone samples (Farr *et al.* 2015), and data for three normal adrenal gland samples were used from datasets GSE88668 and GSE88157. The clinical characteristics and treatment histories of the autopsy cohort have been previously described in detail by Jasu *et al.* (2021) and Sainio *et al.* (2018), and included conventional androgen deprivation therapies, and radiation therapy or prostatectomy in some cases.

For comparison with non-malignant prostate tissue, seven BPH samples were employed (EGAS00001000526). All publicly available RNA-seq data files were downloaded in FASTQ format.

For the prognostic analysis, a previously published prostatectomy cohort consisting of 81 untreated PCa patients (EGAD00001009996) was utilized (Sattari *et al.* 2023). Additional details about this cohort are provided in Supplementary Table S2.

In addition, prostate tissue microarray (TMA) samples of 130 untreated PCas and 34 locally recurrent CRPCs were used for immunohistochemistry (IHC) analysis. Detailed clinical data for the 130 PCa samples used in the survival analysis are provided in Supplementary Table S3. Our previously published RNA-seq data from LNCaP cells were used to address the androgen responsiveness of TMEM18 expression (GSE223024) (Sattari *et al.* 2023).

### Pseudoalignment and expression quantification

Quantification of expression of genes was performed with Kallisto v.0.46.0 (Bray *et al.* 2016). Kallisto transcriptome index was built from the Ensembl Homo_sapiens.GRCh38.109.gtf file.

### Data visualization

To visualize and cluster RNA-seq data in the mCRPC cohort, variance stabilizing transformation was employed, which removes the dependence of the variance on the mean. The vst function of DESeq2 version 1.40.2 was utilized for this purpose (Love *et al.* 2014). PCA plots were then generated using expression values from all annotated genes in the Ensembl Homo_sapiens.GRCh38.109.gtf file. A subset of 32 samples, obtained from the six patients for whom both primary tumor and matched metastasis samples were available, was used in the PCA.

### Metastasis-associated gene discovery pipeline

To identify genes associated with metastasis, we adapted the same approach we used in our previous study (Sattari *et al.* 2023).

#### Analysis of differential expression

The first phase entailed conducting a differential expression analysis using DESeq2 to compare primary PCa with their metastatic samples. The Benjamini–Hochberg method was employed to account for multiple testing and obtain adjusted *P*-values. Genes with an adjusted *P*-value ≤0.1 were considered differentially expressed. This initial stage utilized a less strict criterion to include a broader range of genes for later screening stages.

#### Filtering with tissue-specific controls

To remove genes whose differential expression was affected by their specific tissue location, three different filtering procedures involving tissue-specific controls were utilized. Initially, all primary tumors were compared to each normal tissue outside the prostate to eliminate genes whose reported differences in expression between primary tumors and metastases could be attributed to increased baseline expression in non-prostate tissues. Furthermore, an analysis was conducted to compare all normal samples from various tissues outside the prostate with all primary tumors. The focus was to exclude genes whose differential expression between primary tumors and metastases can be attributed to their generally higher expression in tissues other than the prostate. Third, BPH samples were used as prostate tissue controls. To exclude genes whose reported differential expression between primary tumors and metastases could be attributed to differential expression between BPH and normal tissues, a comparison was made between BPH samples and all other normal tissues combined. In addition, the investigation included an examination of the direction of gene expression change at each step. Genes that did not exhibit opposing changes in expression between controls and primary tumors or BPH samples were kept. DESeq2 was employed for these filtering stages. An adjusted *P*-value of less than 0.01 was chosen as the threshold for statistical significance in the tissue-specific filtering phases.

#### Pairwise analysis

The directions of expression change of the remaining non-tissue-specific DEGs were examined in six patients with both primary and metastatic tumors. This was done by calculating the average expression values of the DEGs in metastatic tumors for each patient and comparing them to the expression value of their corresponding primary tumor from the same patient. In the case of a patient with two primary cancers, the expression values of DEGs in both primary and metastatic tumors were computed independently and then compared. Genes that exhibited expression changes in divergent directions in more than two patients were excluded from the list of DEGs of interest.

### Gene enrichment analysis using DAVID

To analyze DEGs between primary and metastatic PCa, functional enrichment analysis was performed using the DAVID tool, focusing on KEGG pathways and gene ontology (GO) terms, including biological processes and cellular components (Huang *et al.* 2009, Sherman *et al.* 2022).

### Pathway analysis using ingenuity pathway analysis (IPA)

Pathway and regulatory analysis were further conducted using IPA; version 122103623. IPA provided insights into the regulatory networks and potential upstream factors relevant to PCa progression.

### Regulation of differentially expressed genes by transcription factors (TFs)

The ChIP-seq peaks for androgen receptor (AR), FOXA1, and HOXB13 in human prostate tumor tissues were obtained from a publicly accessible dataset for the purpose of analyzing TF-binding sites (GSE56288) (Pomerantz *et al.* 2015). The number of AR, FOXA1, and HOXB13 peaks that were present inside the regulatory regions (−15 kb/+2 kb from the transcription start site (TSS)) of the DEGs were counted. A minimum of one base-pair overlap was required between a peak and the regulatory region.

*Homo sapiens* meta-cluster peaks were downloaded from the Gene Transcription Regulation Database (GTRD) (Yevshin *et al.* 2019). The enrichment analysis was performed to ascertain whether there was an enrichment of binding sites for specific TFs within the regulatory regions (−15 kb/+2 kb from TSS) of DEGs. The one-tailed Fisher’s exact test was performed to determine whether there was an enrichment of binding sites for a certain TF in the regulatory regions of DEGs compared to the regulatory regions of all genes in the dataset. As a statistical cut-off for enriched TFs, an adjusted *P*-value of less than 0.15 was utilized.

### Regulation of genes by androgens

Our previously published RNA-seq data from LNCaP cells treated with siRNA-mediated AR knockdown and dihydrotestosterone (DHT) stimulation were utilized (GSE223024) (Sattari *et al.* 2023). DESeq2 was used to perform differential expression analysis. An adjusted *P*-value of less than 0.05 was used as a threshold for statistical significance. PSA/KLK3 was used as a positive control for androgen responsiveness.

### ATAC-seq analysis

For this study, we utilized ATAC-seq peaks reported in the dataset by Uusi-Mäkelä *et al.* (2020), which includes samples from BPH, untreated primary prostate cancer (PCa), and locally recurrent CRPC (Uusi-Mäkelä *et al.* 2020).

### Survival analysis and multivariate Cox regression analysis

For the Kaplan–Meier survival analysis, patients were classified into high- and low-expression groups, utilizing the third quartile as the threshold. The Cox proportional hazards model was used to investigate the association between progression-free survival (PFS) and the age at diagnosis, PSA levels, Gleason score (GS), and pathologic T status (pT) (Supplementary Table S4). The age variable was divided into two distinct categories: low (referring to those under the age of 62) and high (referring to individuals above the age of 62). Similarly, the pathologic T status was classified into two categories: low (pT = pT2a-b) or high (pT = pT3a-b). The diagnostic PSA values were categorized into three groups: low (PSA ≤10), intermediate (PSA range from 10 to 19.9), and high (PSA ≥20). The Gleason scores were categorized into three groups: low (GS < 7), intermediate (GS = 7), and high (GS > 7). Four samples were excluded from this analysis due to the absence of Gleason score values.

### Immunohistochemical staining

TMEM18 protein expression levels in prostate carcinomas were determined with IHC using formalin-fixed, paraffin-embedded (FFPE) tissue microarray (TMA) samples. IHC staining for TMEM18 was conducted with the autostainer, employing Nichirei Histofine DAB-2V (Nichirei Biosciences, Japan) and an anti-TMEM18 antibody (HPA040233-100UL, UNSPSC Code: 12352203) at a 1:100 dilution. Negative control staining was performed by omitting the primary antibody to confirm specificity. The slides were then scanned using a NanoZoomer S60 Digital slide scanner (C13210-01, Hamamatsu Photonics, K. K, Japan) with a 20× objective. Cytoplasmic staining intensities of TMEM18 were evaluated on a scale from 0 to 3, where 0 indicated negative staining, 1 weak, 2 moderate, and 3 indicated strong staining within cancerous areas.

## Results

### Prostate cancer metastases exhibit significant variability in gene expression

**Figure 1:**
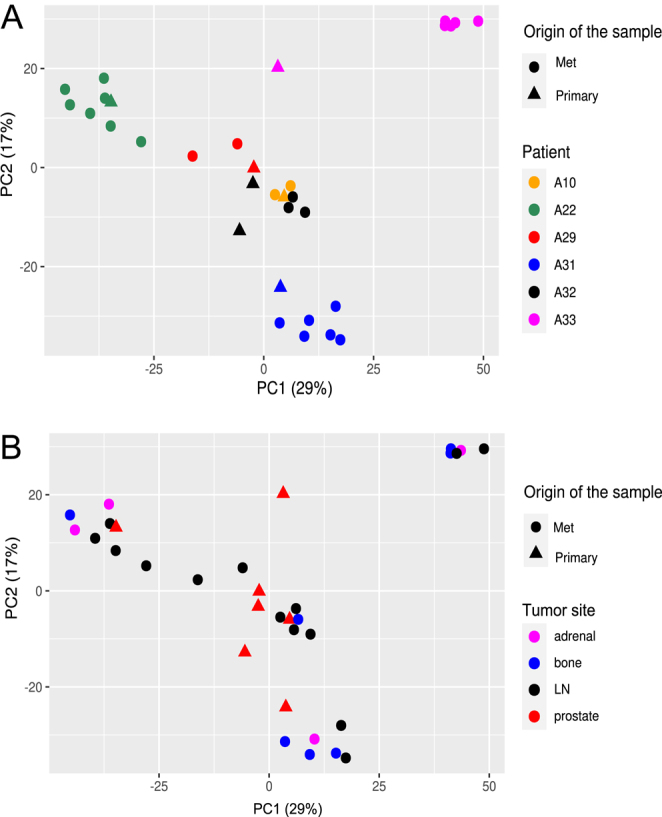
Principal component analysis (PCA) of primary and metastatic tumors. Patients with samples from both their primary tumors and metastases were selected for PCA visualization. RNA-seq data from all annotated genes in the annotation file were included in the PCA, with different visual emphasis on sample characteristics in panels A and B. (A) PCA plot highlighting patient-specific differences and the origin of samples (primary tumors vs metastases). Colors represent individual patients and shapes indicate the origin of samples. (B) PCA plot emphasizes tumor sites and the origin of samples. Colors represent tumor sites and shapes indicate the origin of samples (primary vs metastatic). The percentages of variance explained by principal components 1 and 2 are indicated on the respective axes. A full color version of this figure is available at https://doi.org/10.1530/ERC-25-0070.

To pinpoint genes specifically expressed in mCRPC, we compared the expression patterns of all genes between samples of primary prostate tumors and metastases. First, we evaluated the collective impact of various variables, including origin of the sample (primary or metastatic tumor), patient ID, and the tumor site, on gene expression. Principal component analysis (PCA) was performed using a subset of gene expression data consisting of samples from patients who had both primary tumors and metastases (Fig. 1). These analyses indicated that the key factor influencing clustering was patient identity, while there was no observable clustering based on whether the sample originated from a primary tumor or metastasis, or the specific site of metastasis (Fig. 1). The results suggest that most of the variance in gene expression within primary tumors and metastatic PCa samples originates from variance between patients.

### Discovering genes associated with prostate cancer metastasis

**Figure 2:**
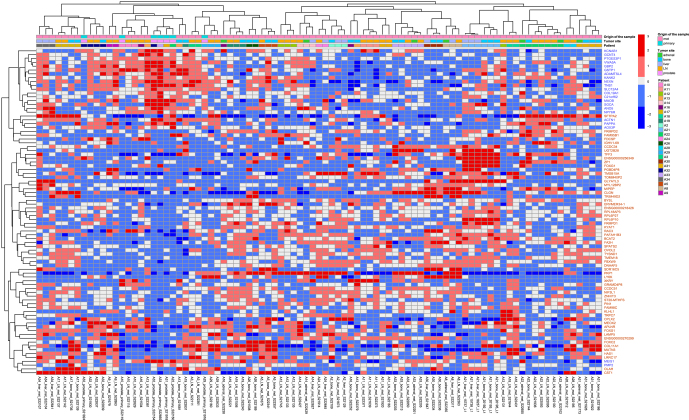
Heatmap of DEGs between primary and metastatic tumors. Heatmap showing the expression levels of DEGs across the samples. The color bars at the top represent the different variables in the dataset, patient numbers, tumor sites, and origin of the samples. The expression value of each DEG is depicted using a color scale, with red indicating a high relative expression and blue indicating a low relative expression. The DEGs were clustered based on their expression patterns across all samples. The genes are color-coded according to their upregulation (red) or downregulation (blue). A full color version of this figure is available at https://doi.org/10.1530/ERC-25-0070.

The differential expression analysis comparing primary PCa to metastatic lesions yielded 779 DEGs with an adjusted *P*-value below 0.1. Among these, 572 genes exhibited significant upregulation, while 207 showed downregulation in the metastases compared to the primary tumors. It has been shown that the expression levels of protein-coding genes can vary significantly depending on the site of expression (Fagerberg *et al.* 2014). Therefore, to determine whether the variation in expression of the identified DEGs in metastatic sites was associated with specific tissue types, we investigated the tissue specificity of our DEGs through a three-step process outlined in the methods. Following the tissue-specific filtering, 91 DEGs remained, with 67 being upregulated and 24 being downregulated. As a significant amount of patient-to-patient variability was found in DEGs, we performed a paired analysis for the remaining non-tissue-specific DEGs to lessen the effects of this variability, resulting in a set of 85 DEGs, comprising 63 upregulated and 22 downregulated genes. A heatmap displaying the expression of the 85 DEGs is presented in Fig. 2. Gene clustering was conducted based on the expression patterns of the DEGs in these samples. While patient IDs mostly impacted sample clustering, some of the primary tumor samples clustered together, indicating a degree of similarity. In addition, there was evidence of clustering within patients based on tissue specificity (Fig. 2).

### Enrichment analysis highlights key pathways in prostate cancer

Functional enrichment analysis of the identified 85 genes, which were differentially expressed between primary and metastatic PCa, was performed using DAVID tool (DAVID Functional Annotation Bioinformatics Microarray Analysis) (Sherman *et al.* 2022), focusing on KEGG pathways, gene ontology biological processes, and cellular components. The results illustrate associations between the genes and enriched biological processes, such as extracellular matrix organization and collagen-containing extracellular matrix, which are crucial for cancer cell adhesion, invasion, and metastasis (Supplementary Table S5). Metabolic pathways and drug metabolism, particularly the cytochrome P450 system, suggest potential mechanisms of metabolic reprogramming and drug resistance in PCa (Supplementary Table S5).

Further functional analysis of the 85 DEGs was conducted using IPA, providing insights into the key molecular pathways and upstream regulators relevant to PCa (Supplementary Table S6). IPA identified ‘invasive cancer’ as a significantly enriched disease annotation highlighting genes such as FOXC2, GSTP1, RAC3, PAPPA, and COL11A1. These genes are involved in critical processes such as epithelial-to-mesenchymal transition (EMT), cell migration, extracellular matrix remodeling, and resistance to therapeutic agents, all of which contribute significantly to cancer cell invasiveness and metastatic potential (Børretzen *et al.* 2019, Nallanthighal *et al.* 2021, Zheng *et al.* 2021, Aykanli *et al.* 2024, Yu *et al.* 2025).

The upstream regulator analysis from IPA further revealed transcriptional regulators significantly associated with our gene set. Notably, MECP2 and ARID1A emerged as important regulators, both known to influence chromatin remodeling and transcriptional regulation in PCa (Li *et al.* 2022, Yang *et al.* 2023). In addition, IPA identified a transcriptional regulatory complex comprising TFF3, FOXA1, GATA3, ESR1, and EP300, emphasizing hormone-responsive and epithelial differentiation-related pathways critical to PCa progression and castration resistance. Together, these findings elucidate the underlying regulatory networks and invasive pathways potentially contributing to aggressive PCa phenotypes (Nguyen *et al.* 2013, Jia *et al.* 2017, Welti *et al.* 2021) (Supplementary Table S6).

### Exploring transcription factors controlling gene expression in prostate cancer metastasis

Elevated AR expression is a significant contributor to the progression of PCa to CRPC (Visakorpi *et al.* 1995, Linja *et al.* 2001). The AR pioneering factor FOXA1, together with HOXB13, is also involved in this process (Pomerantz *et al.* 2015). Therefore, we investigated the AR, FOXA1, and HOXB13 binding in the regulatory regions (−15 kb/+2 kb from TSS) of the DEGs by utilizing previously published prostate tumor and prostate normal tissue-specific AR-binding sites (ARBSs) and FOXA1- and HOXB13-binding sites in PCa tumor samples (Pomerantz *et al.* 2015).

**Figure 3:**
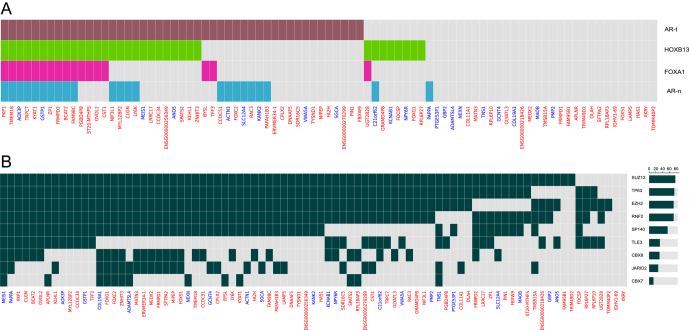
TFs enriched in the regulatory regions of DEGs. (A) The heatmap depicts the presence of binding sites for AR, FOXA1, and HOXB13 within the regulatory regions of DEGs. Each colored bar indicates a binding event for the corresponding TF across DEGs. Gene names are color-coded by their direction of regulation: red for upregulated, blue for downregulated DEGs. (B) The heatmap displays the presence of binding sites for the nine enriched TFs within the regulatory regions (−5 kb/+2 kb TSS) of DEGs. These binding sites were identified using ChIP-seq peaks from the GTRD database. The dark red color corresponds to the presence of binding sites for each TF, whereas the light gray tint indicates the absence of binding sites. The right side of the heatmap represents the count of DEGs that have a binding site for the specified TF. A full color version of this figure is available at https://doi.org/10.1530/ERC-25-0070.

Results showed that among the 85 DEGs, 47 genes had tumor-specific ARBSs, but only 23 genes had normal sample-specific ARBSs (Fig. 3A). Fourteen of the 47 prostate tumor-specific ARBSs (Fig. 3A) were co-occupied by both FOXA1 and HOXB13, implying that FOXA1 and HOXB13 also contribute to the regulation of these DEGs in PCa.

To learn more about the regulatory mechanism of the DEGs, we performed TF enrichment analysis using the GTRD database to find out which TF-binding sites were enriched in the promoter region of our metastasis-associated genes. With an adjusted *P*-value below 0.1, we identified nine TFs named EZH2, SUZ12, TLE3, TP63, CBX7, RNF2, SP140, JARID2, and CBX8, whose binding sites were enriched in the regulatory regions of the 85 DEGs (Fig. 3B). Prior studies have linked most of the discovered TFs to PCa. We next assessed the expression profiles of a selected set of TFs in primary tumors and mCRPC samples to evaluate their potential involvement in metastatic progression. As shown in Supplementary Fig. 1, several TFs, such as TLE3, SUZ12, and CBX7, were expressed at relatively high levels across the cohort. While the expressions of CBX7 and TP63 were noticeably higher in primary tumors, TLE3 and SP140 showed elevated levels in metastatic samples. This variation in expression patterns may reflect functional differences in the transcriptional programs between primary and metastatic tumors.

### Association between differentially expressed genes and progression-free survival in prostatectomy-treated patients

**Figure 4:**
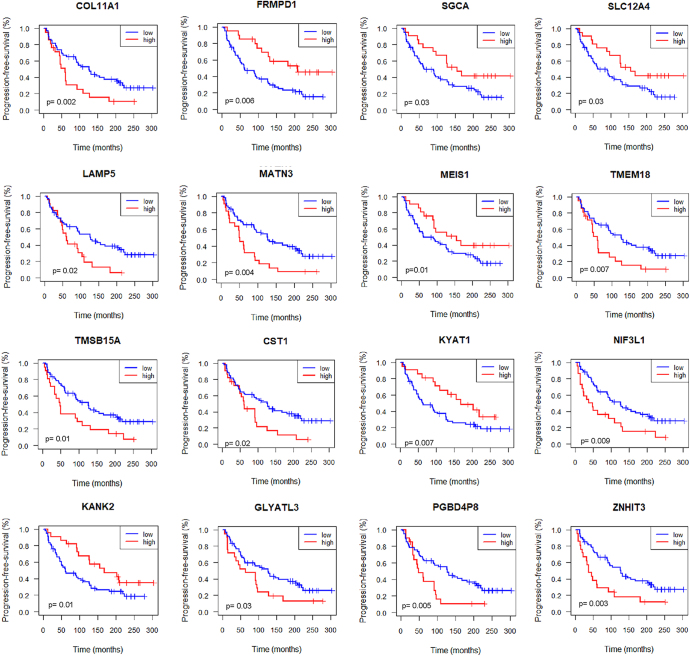
Survival analysis of the prostatectomy cohort. Kaplan–Meier analysis was employed to examine the PFS in patients with PCa using RNA expression data. The third quartile was utilized as a threshold to categorize expression levels of DEGs into high and low. *P*-values were calculated by log-rank test. A full color version of this figure is available at https://doi.org/10.1530/ERC-25-0070.

Next, to explore the prognostic potential of the metastasis-associated DEGs, we determined their expression in our prostatectomy cohort and examined their association with PFS. The Kaplan–Meier survival analysis was used to compare patients with high and low expression levels of our 85 DEGs. With a *P*-value less than 0.05, we observed that the expression of 16 DEGs, namely COL11A1, CST1, FRMPD1, GLYATL3, KANK2, KYAT1, LAMP5, MATN3, MEIS1, NIF3L1, PGBD4P8, SGCA, SLC12A4, TMEM18, TMSB15A, and ZNHIT3, showed a significant association with PFS (Fig. 4). Specifically, among the 12 genes upregulated in mCRPC, higher expression of 11 genes (COL11A1, LAMP5, MATN3, TMEM18, TMSB15A, CST1, KYAT1, NIF3L1, GLYATL3, PGBD4P8, and ZNHIT3) correlated with significantly worse PFS, consistent with their association with aggressive disease. FRMPD1, despite being upregulated, was an exception, showing an association with improved survival. Conversely, among the four downregulated genes (KANK2, SGCA, SLC12A4, and MEIS1), higher expression correlated with better prognosis, suggesting their potential tumor-suppressive roles.

In addition, we employed a multivariate Cox regression analysis to determine the independence of these 16 DEGs as prognostic markers. The expression levels of FRMPD1, TMEM18, and ZNHIT3 were independent predictors for biochemical recurrence (*P*-value <0.05) (Table 1). In addition, the associations between the expression levels of TMEM18, FRMPD1, and ZNHIT3 with clinical parameters, including age, Gleason score (GS), diagnostic PSA levels, and pathological T stage (pT), were analyzed. TMEM18 showed a significant association with diagnostic PSA levels (*P* < 0.01), but no significant association with age, GS, or pT. FRMPD1 did not exhibit a significant association with any of these parameters. In contrast, ZNHIT3 expression was significantly associated with age (*P* < 0.05) and diagnostic PSA levels (*P* < 0.01), but showed no significant associations with GS or pT (Supplementary Fig. 2).

**Table 1:** Multivariate Cox regression analysis.

Variable	TMEM18		ZNHIT3		FRMPD1	
	RR (95%CI)	*P*-value	RR (95%CI)	*P*-value	RR (95%CI)	*P*-value
Low expression	1.000 (reference)		1.000 (reference)		1.000 (reference)	
High expression	2.056 (1.125–3.758)	0.019	2.092 (1.158–3.779)	0.014	0.458 (0.221–0.948)	0.035
PSA ≤10	1.000 (reference)		1.000 (reference)		1.000 (reference)	
PSA from 10 to 19.9	1.251 (0.650–2.406)	0.50	1.200 (0.622–2.314)	0.585	1.456 (0.718–2.949)	0.297
PSA >20	4.030 (1.763–9.207)	0.0009	3.559 (1.558–8.131)	0.002	3.922 (1.723–9.924)	0.001
GS < 7	1.000 (reference)		1.000 (reference)		1.000 (reference)	
GS = 7	1.566 (0.795–3.083)	0.194	1.476 (0.762–2.856)	0.306	1.114 (0.524–2.110)	0.886
GS > 7	2.486 (1.153–5.355)	0.020	2.251 (1.047–4.834)	0.037	2.026 (0.943–4.351)	0.070
Age ≤62	1.000 (reference)		1.000 (reference)		1.000 (reference)	
Age >62	1.476 (0.828–2.630)	0.186	1.544 (0.864–2.759)	0.142	1.381 (0.751–2.537)	0.297
pT = pT2a-b	1.000 (reference)		1.000 (reference)		1.000 (reference)	
pT = pT3a-b	1.747 (1.022–2.984)	0.041	1.701 (0.990–2.921)	0.054	1.651 (0.951–2.864)	0.074

The third quartile of gene expression level was used as the threshold to differentiate between high and low gene expression levels.

The average age of the patients was 62 years.

RR (relative risk), pT (pathological T stage).

### ATAC-seq analysis

We then evaluated the chromatin accessibility in the promoter regions of FRMPD1, TMEM18, and ZNHIT3 using ATAC-seq data from clinical patient samples from BPH, untreated primary PCa, and locally recurrent CRPC samples. The results indicate open chromatin in the promoter region of FRMPD1 and TMEM18 across all samples. An ATAC-seq peak was not found in the promoter region of ZNHIT3 (Supplementary Fig. 3). In addition to the ATAC-seq peaks, we included AR ChIP-seq peaks in human prostate tumor tissues retrieved from a publicly available dataset (GSE56288) and ChIP-seq peaks for TFs associated with PCa in the GTRD database (Supplementary Fig. 3).

Since the results indicated that there is an ARBS in TMEM18, we examined the influence of androgens on TMEM18. For this, we analyzed TMEM18 expression using RNA-seq in LNCaP cells treated with either 0 nM or 10 nM DHT. The data indicated a minor but significant upregulation for TMEM18 in LNCaP cells following DHT stimulation (Supplementary Table S7). We then showed the expression levels of TMEM18 in BPH, primary tumors, and metastatic samples, and in different tissue types (prostate, adrenal, liver, bone, and lymph node). As illustrated in Supplementary Fig. 4A, TMEM18 expression is significantly elevated in metastatic samples compared to both primary tumors and normal prostate samples (Supplementary Fig. 4A). In addition, among different tissue types, metastases exhibit the highest levels of TMEM18 expression, suggesting a potential role in advanced disease progression (Supplementary Fig. 4B).

### Immunohistochemical analysis

**Figure 5:**
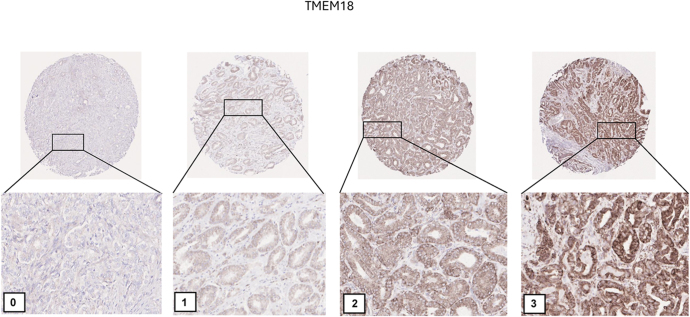
Immunohistochemical (IHC) analysis of TMEM18 expression in PCa specimens. Representative IHC staining images of PCa specimens for TMEM18 expression are shown. The staining intensity is categorized into four levels, labeled directly in the images: 0 (negative), 1 (low), 2 (moderate), and 3 (high). The images demonstrate strong cytoplasmic positivity for TMEM18, with increasing intensity corresponding to higher expression levels. Enlarged views below each core highlight the staining patterns and differences in expression intensity across the specimens. A full color version of this figure is available at https://doi.org/10.1530/ERC-25-0070.

**Figure 6:**
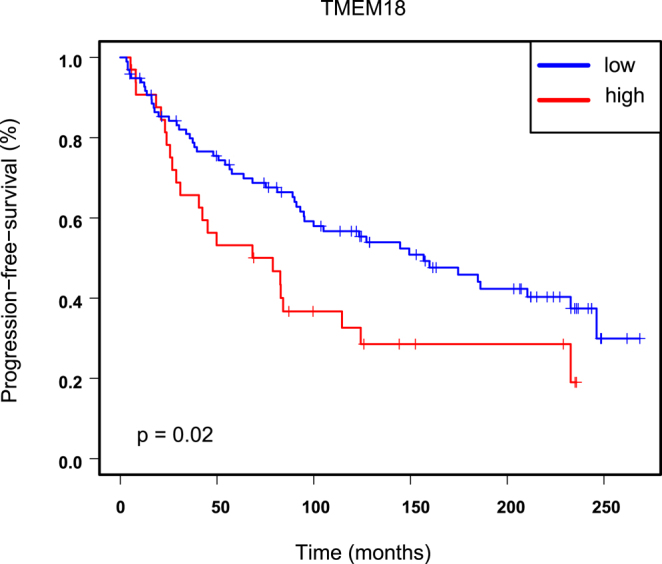
Immunohistochemistry analysis of TMEM18 expression in PCa. Kaplan–Meier analysis was used to evaluate PFS in patients with PCa. Patients were grouped based on TMEM18 protein expression levels determined by immunohistochemistry. Immunohistochemistry intensity levels 0, 1 and 2 were categorized as low, and intensity level 3 was considered high. *P*-values were determined using the log-rank test. A full color version of this figure is available at https://doi.org/10.1530/ERC-25-0070.

Next, immunohistochemical (IHC) analysis of PCa specimens was performed for TMEM18. The IHC in PCa specimens showed strong cytoplasmic staining, with intensity levels categorized as 0, 1, 2, and 3 (Fig. 5). The IHC data revealed a significant association between TMEM18 expression and PFS, with high TMEM18 expression associated with shorter PFS (*P* = 0.022) (Fig. 6).

Furthermore, multivariate analysis identified TMEM18 staining as an independent prognostic marker (Table 2). The association between TMEM18 staining and clinical parameters, such as age, GS, diagnostic PSA levels, and pT, is shown (Supplementary Fig. 5). TMEM18 expression does not demonstrate significant associations with GS, age, PSA levels, or pT categories (all *P* > 0.05). However, IHC analysis reveals a significant increase in TMEM18 expression from primary PCa to CRPC, suggesting a potential role for TMEM18 in the progression to a more aggressive disease state (Supplementary Fig. 6).

**Table 2:** Multivariate Cox regression analysis.

TMEM18	RR (95% CI)	*P*-value
variable		
Low intensity	1.000 (reference)	
High intensity	1.9587 (1.134–3.382)	0.015
PSA ≤10	1.000 (reference)	
PSA from 10 to 19.9	1.8863 (1.018–3.494)	0.043
PSA >20	2.6745 (1.356–5.273)	0.004
GS < 7	1.000 (reference)	
GS = 7	1.2280 (0.675–2.232)	0.50
GS > 7	0.8742 (0.327–2.337)	0.788
Age ≤62	1.000 (reference)	
Age >62	0.9648 (0.588–1.582)	0.887
pT = pT2a-b	1.000 (reference)	
pT = pT3a-b	1.8423 (1.084–3.129)	0.023

Immunohistochemistry (IHC) staining intensity was used as the threshold to differentiate between high and low protein expression levels. Intensity levels 1 and 2 were classified as low, and intensity level 3 was classified as high expression level.

The average age of the patients was 62 years.

RR (relative risk), pT (pathological T stage).

## Discussion

While most newly diagnosed PCa cases are slowly growing, some of them can become aggressive and potentially lethal. Hence, identifying biomarkers indicating the probability of developing metastatic PCa is crucial. Our study addresses this need by utilizing a unique dataset encompassing samples from both primary tumors and castration-resistant metastases, the latter presenting the most aggressive form of PCa.

The results here show that the variations in protein-coding gene expression within primary tumors and metastatic PCa samples are largely due to variances between patients, as we have previously shown to be the case with lncRNA expression (Sattari *et al.* 2023). This demonstrates a significant role of the tumor genome in gene expression in metastases. We have previously shown with this same cohort that the genetic changes are mostly the same between the metastases and the primary tumors of corresponding patients, while there is plenty of variation in the genetic alterations between patients (Liu *et al.* 2009, Gundem *et al.* 2015). We used a variety of methods to reduce the impacts of patient differences and tissue types. As a result, we successfully identified 85 metastasis-specific DEGs. Of these, 63 were transcriptionally upregulated and 22 were downregulated in the metastases.

To understand the biological pathways associated with these DEGs, functional enrichment analysis was conducted, focusing on KEGG pathways, gene ontology biological processes, and cellular components. These analyses underscored the involvement of key processes, such as extracellular matrix organization and collagen-containing extracellular matrix, both of which are essential for cancer cell adhesion, motility, and invasiveness. These processes are directly relevant to metastatic potential in PCa, highlighting the role of the cytoskeleton and extracellular matrix in the disease’s progression and metastatic capacity. Further functional insights from IPA linked our DEGs to invasive cancer pathways and identified upstream regulators such as ARID1A and MECP2, which play established roles in PCa progression (Bernard *et al.* 2006, Li *et al.* 2022).

The primary TFs extensively studied in PCa, including AR (Fujita & Nonomura 2019) and its well-established co-regulators FOXA1 and HOXB13, play a significant role in driving the disease (Pomerantz *et al.* 2015). In our study, specific analysis of publicly available prostate tumors and normal tissue-specific AR-binding sites (ARBSs) revealed that among the 85 DEGs, 47 had at least one tumor-specific AR-binding site within their regulatory regions. In contrast, only 23 out of the 85 DEGs contained at least one normal-specific AR-binding site within their regulatory regions. Furthermore, we found the co-occupation of AR, FOXA1, and HOXB13 in the regulatory regions of 14 out of the 85 DEGs. To obtain more insight into the putative transcriptional regulators of the DEGs, we applied TFEA using the GTRD database. The analysis showed that the regulatory areas of our DEGs were enriched with nine TFs, namely EZH2, SUZ12, CBX7, CBX8, TP63, RNF2, SP140, JARID2, and TLE3. Previous research has indicated associations between EZH2, SUZ12, CBX7, TP63, RNF2, JARID2, and TLE3 and PCa (Parsons *et al.* 2009, Crea *et al.* 2011, Kim & Roberts 2016, Wei *et al.* 2016, Palit *et al.* 2019, Gong *et al.* 2020, Zhang *et al.* 2022). EZH2 and SUZ12 are critical components of the polycomb repressive complex 2 (PRC2), which is known to mediate gene silencing through histone methylation, highlighting their role in epigenetic regulation in PCa (Margueron & Reinberg 2011, Rai *et al.* 2013). Similarly, the enrichment of CBX7 and CBX8, both key components of PRC1, underscores their role in the epigenetic regulation of genes implicated in PCa. CBX7 has been linked to the repression of tumor suppressor genes, contributing to cancer progression and poor prognosis. In contrast, CBX8 exhibits a more dynamic function, participating in both transcriptional repression and activation, depending on the context (Wang *et al.* 2023, Wanli *et al.* 2023, Xu *et al.* 2024).

Since AR-binding sites were found in the regulatory areas of most genes, not just the DEGs, the results did not reveal enrichment of AR within the regulatory regions of these 85 DEGs. Kaplan–Meier analysis of the prostatectomy-treated men identified 16 DEGs, namely COL11A1, FRMPD1, SGCA, SLC12A4, LAMP5, MATN3, MEIS1, TMEM18, TMSB15A, CST1, KYAT1, NIF3L1, KANK2, GLYATL3, PGBD4P8 and ZNHIT3, to possess prognostic value. Out of those, Cox multivariate regression analysis identified three genes (FRMPD1, TMEM18, and ZNHIT3) as independent predictors of biochemical recurrence. TMEM18 was found to be androgen-responsive in LNCaP cells. Further validation through IHC analysis confirmed that TMEM18 expression is significantly correlated with PFS, both at the protein and RNA levels. TMEM18 is a transmembrane protein that has been implicated in various cellular processes, including cell migration and adhesion, and is located in the nuclear membrane (Jurvansuu & Goldman 2011). The prognostic potential of TMEM18 has been recognized in other malignancies, including acute myeloid leukemia (AML) (Ha *et al.* 2018), highlighting its clinical significance. In AML patients, particularly males, overexpression of TMEM18 is linked to a more favorable prognosis. However, its significance in PCa has previously been unexplored.

In summary, our findings highlight potential genes that could contribute to the progression of PCa and potentially serve as biomarkers for aggressive disease with poor prognosis. Future research is warranted to evaluate the potential role of TMEM18 in promoting metastasis and the clinical relevance of its prognostic potential in the context of PCa.

## Supplementary materials

















## Declaration of interest

The authors declare that there is no conflict of interest that could be perceived as prejudicing the impartiality of the work reported.

## Funding

This research was supported by the Translational Research Network for Prostate Cancer (TransPot) and was funded by the European Union’s Horizon 2020 research and innovation programme under the Marie Skłodowska-Curie grant agreement no 721746 (TV). In addition, this research was supported by the Academy of Finland (TV 279270; LL 357490; MN 312043; and GSB 294820), the Sigrid Juselius Foundation (TV, GSB, and MN), the Cancer Foundation Finland (TV, LL, GSB, and MN), and the Competitive State Research Financing of the Expert Responsibility area of Tampere University Hospital (TV).

## Author contribution statement

MS took part in the project design, was primarily responsible for data generation and analysis, and prepared the initial draft of the manuscript. HR contributed to the project design, data analysis, and manuscript preparation. WBI provided the necessary samples and assisted with manuscript preparation. MN participated in the study design, data analysis, and manuscript preparation. TV, JK and LL were involved in the project design, data analysis, manuscript preparation, and overall supervision of the project’s progress. All authors contributed to editing of the manuscript and approved the final version for publication.

## Data availability

RNA-seq data for metastatic prostate tissues (from liver, adrenal gland, bone, subdural, and lymph node) and matched normal liver/adrenal samples are available in the European Genome-phenome Archive (EGA) under accession number EGAS00001006959. Additional RNA-seq data for normal bone were obtained from GSE72815, and normal adrenal gland data from GSE88668 and GSE88157. Benign prostatic hyperplasia (BPH) samples are available under EGAS00001000526. AR ChIP-seq peaks in human prostate tumor tissues were retrieved from GSE56288, and additional TF ChIP-seq data for PCa-related TFs were sourced from the GTRD database (see Supplementary Fig. 2 for details). All datasets are publicly accessible under the cited accession numbers.

## Ethics approval and consent to participate

This study involving human prostate cancer specimens received approval from the Ethics Committee of Tampere University Hospital and the National Authority for Medicolegal Affairs (ETL code R03203). Informed consent was obtained from all participants. The specimens were de-identified, and all procedures involving human samples were conducted following the relevant ethical guidelines and regulations. Autopsy tissue samples were collected as part of the PELICAN integrated clinical-molecular autopsy study of lethal prostate cancer. The patients included in this study consented to participate in the John Hopkins Medicine IRB-approved study (NA_00003925) between 1995 and 2005.
