# Supplementary material for: Identification of protein-coding genes associated with metastatic prostate cancer
Source: Endocr Relat Cancer. 2025 Jun 26;32(7):e250070. doi: 10.1530/ERC-25-0070 (PMC12203752; doi:10.1530/ERC-25-0070)
Supplement: Supplementary file 8 [file Supplmentary_Figures_S1-S6.pdf]

Supplementary Figures

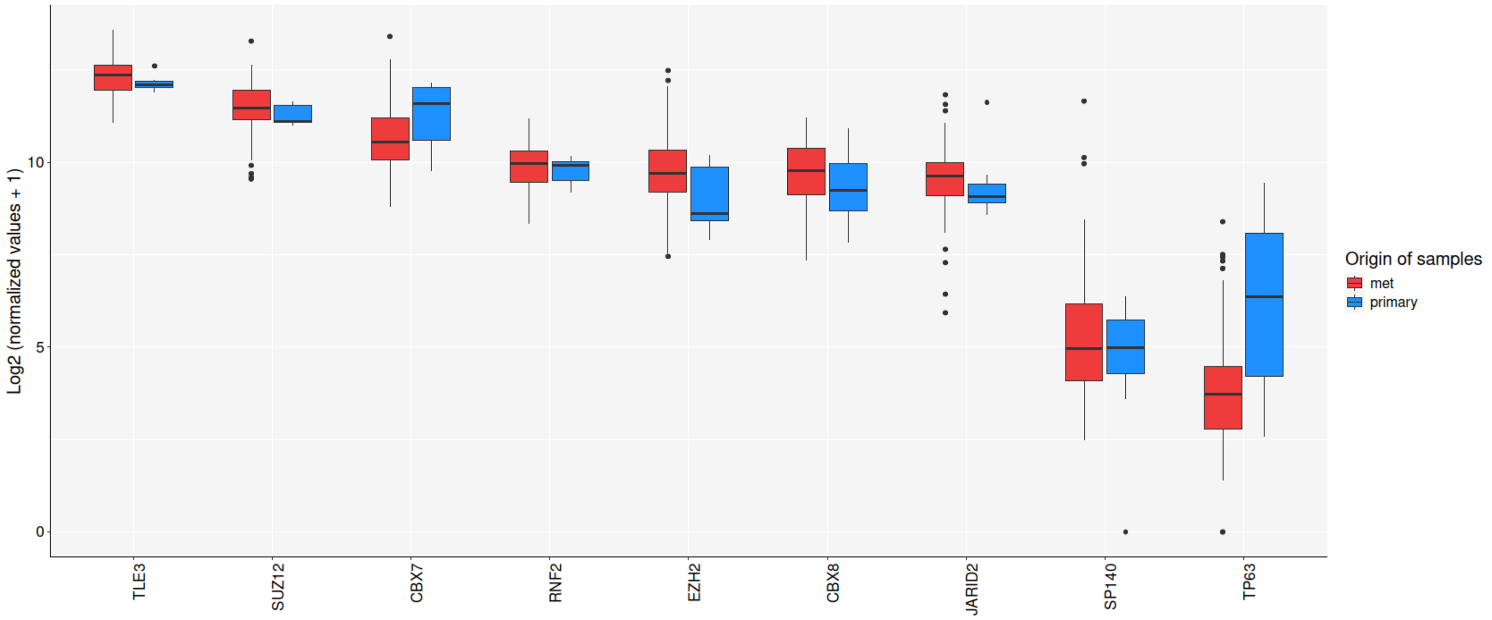

Supplementary Fig. 1. Expression profiles of selected transcription factors in primary and metastatic PCa samples.

Gene expression values were normalized using the DESeq2 method. Box plots are presented on a log2 (Deseq2 normalized values + 1) scale.

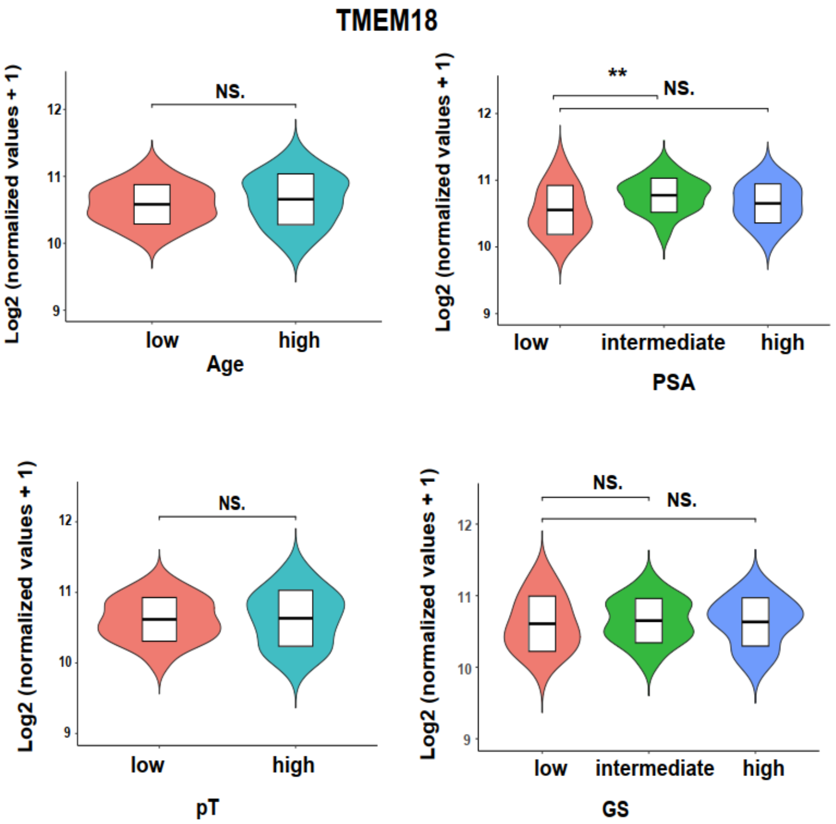

FRMPD1

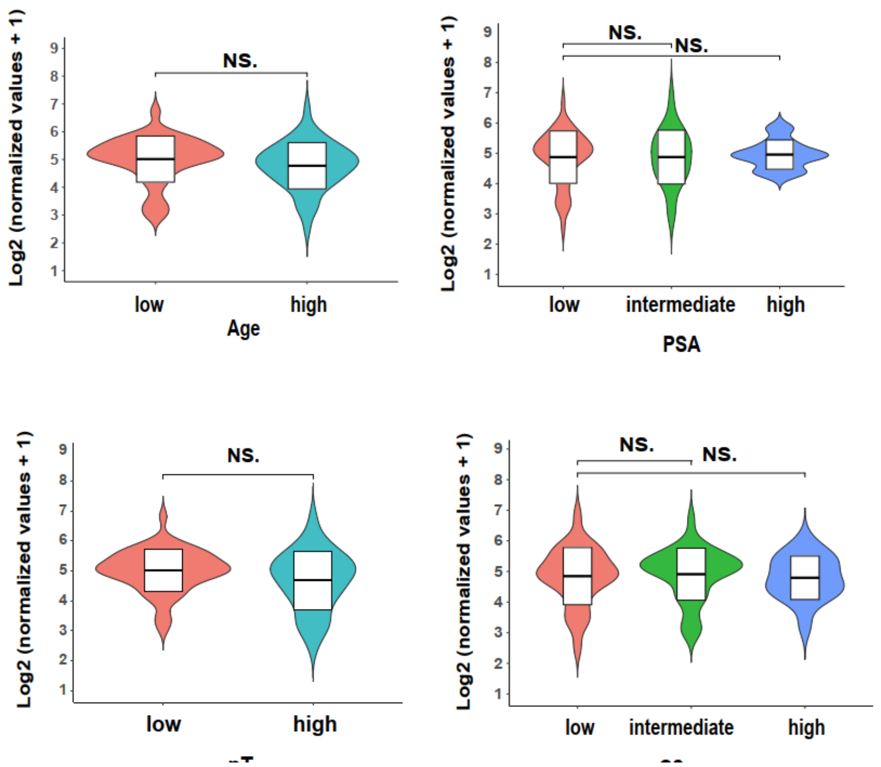

ZNHIT3

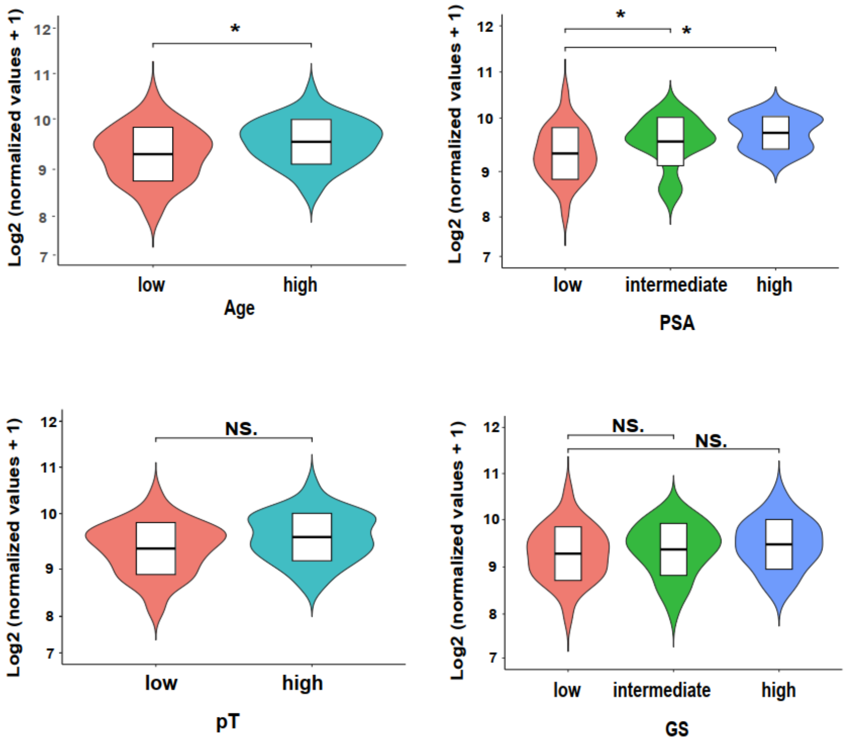

Supplementary Fig. 2. Correlations between the RNA expression levels of TMEM18, FRMPD1 and ZNHIT3 and clinical parameters. Violin plots showing the expression levels (Log2 normalized values + 1) of TMEM18, FRMPD1 and ZNHIT3 in relation to clinical parameters: Age (low vs. high), PSA (low, intermediate, high), pT (low vs. high), and Gleason Score (GS) (low, intermediate, high). Boxes within the plots indicate the interquartile range and median. Statistical significance is denoted as NS (not significant), * ($p < 0.05$), and ** ($p < 0.01$).

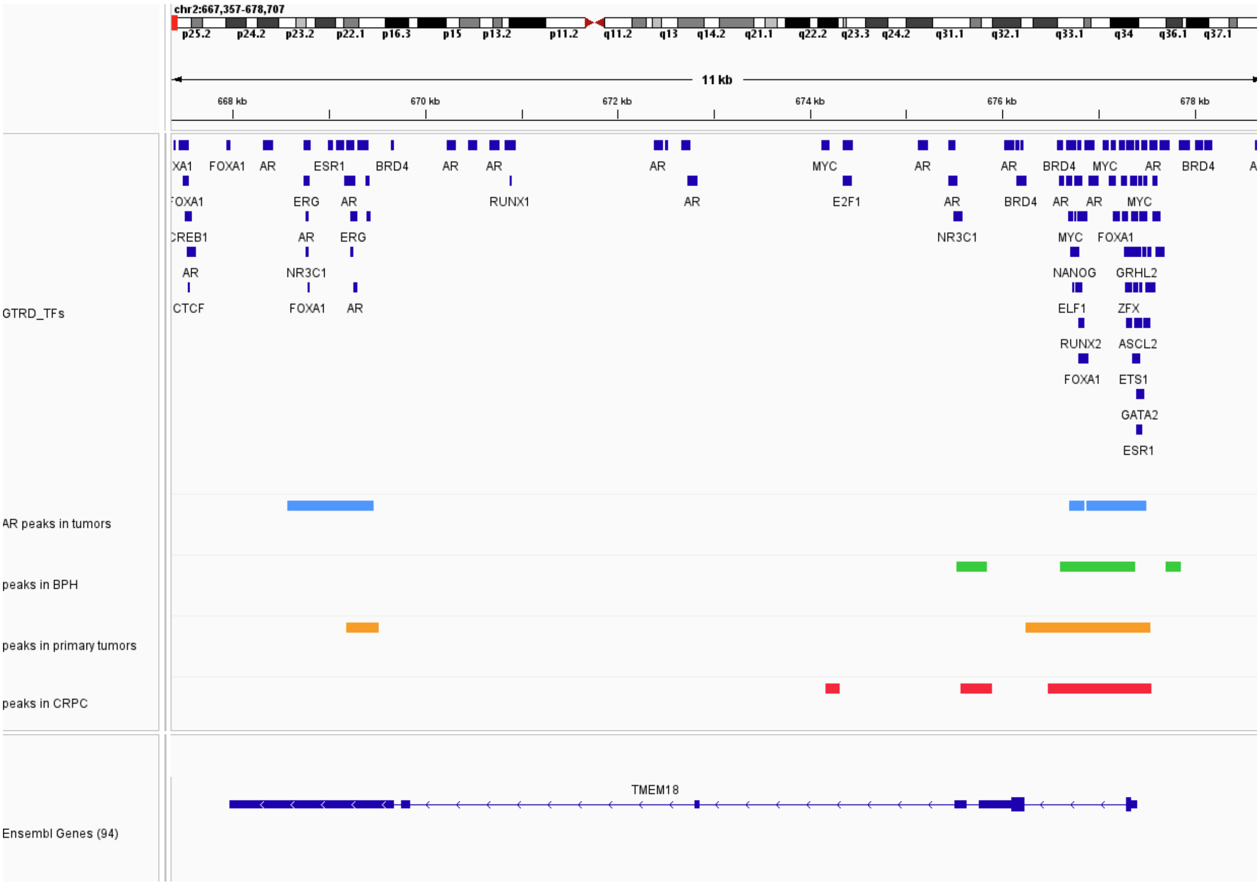

chr2:667,357-678,707
p25.2 p24.2 p23.2 p22.1 p16.3 p15 p13.2 p11.2 q11.2 q13 q14.2 q21.1 q22.2 q23.3 q24.2 q31.1 q32.1 q33.1 q34 q36.1 q37.1
11 kb
668 kb 670 kb 672 kb 674 kb 676 kb 678 kb
GTRD_TFs
FOXA1 AR ESR1 BRD4 AR AR AR MYC AR AR BRD4 MYC AR BRD4
ERG AR RUNX1 AR E2F1 AR BRD4 AR AR MYC
AR ERG NR3C1 MYC FOXA1
AR NR3C1 NANOG GRHL2
CTCF FOXA1 AR ELF1 ZFX
RUNX2 ASCL2
FOXA1 ETS1
GATA2
ESR1
AR peaks in tumors
peaks in BPH
peaks in primary tumors
peaks in CRPC
Ensembl Genes (94)
TMEM18

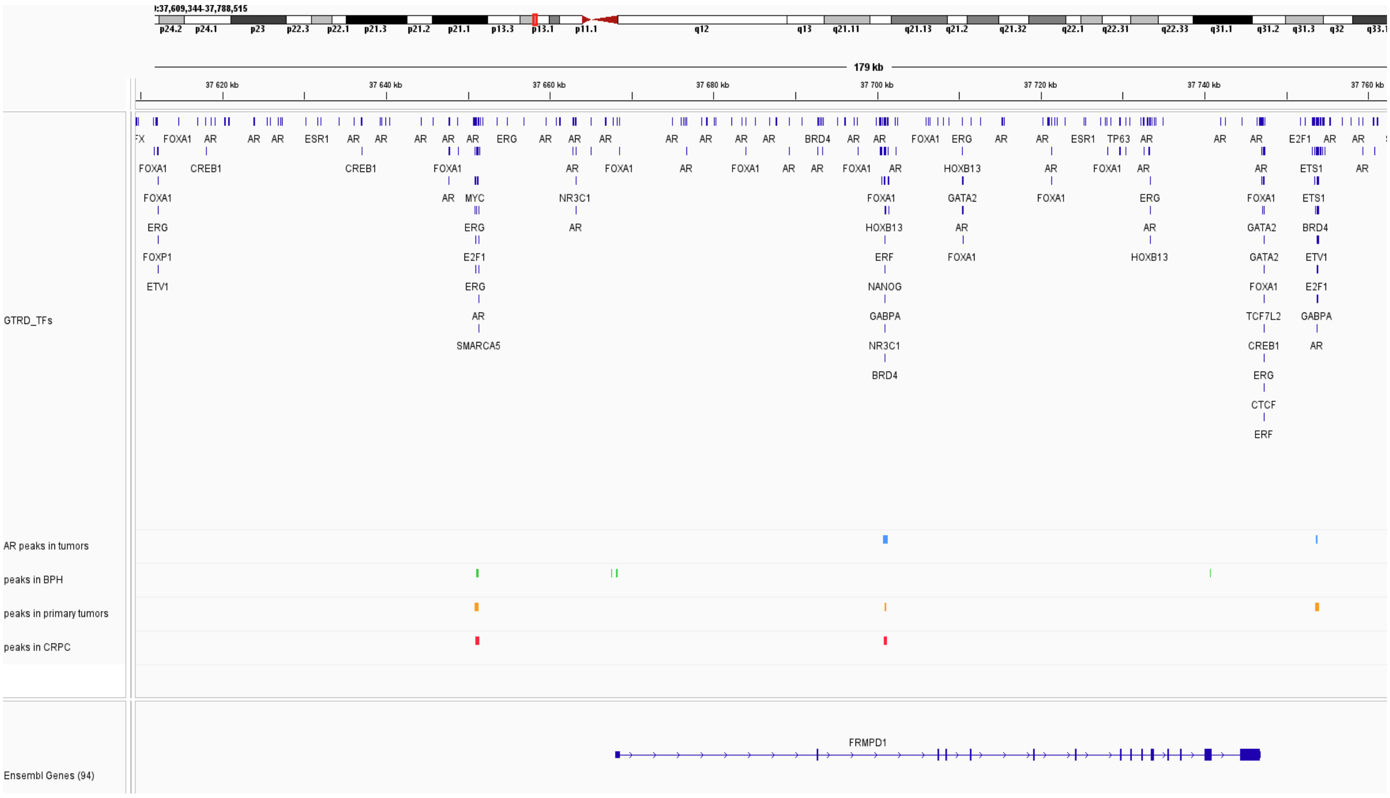
179 kb
GTRD_TFs
AR peaks in tumors
peaks in BPH
peaks in primary tumors
peaks in CRPC
Ensembl Genes (94)
FRMPD1

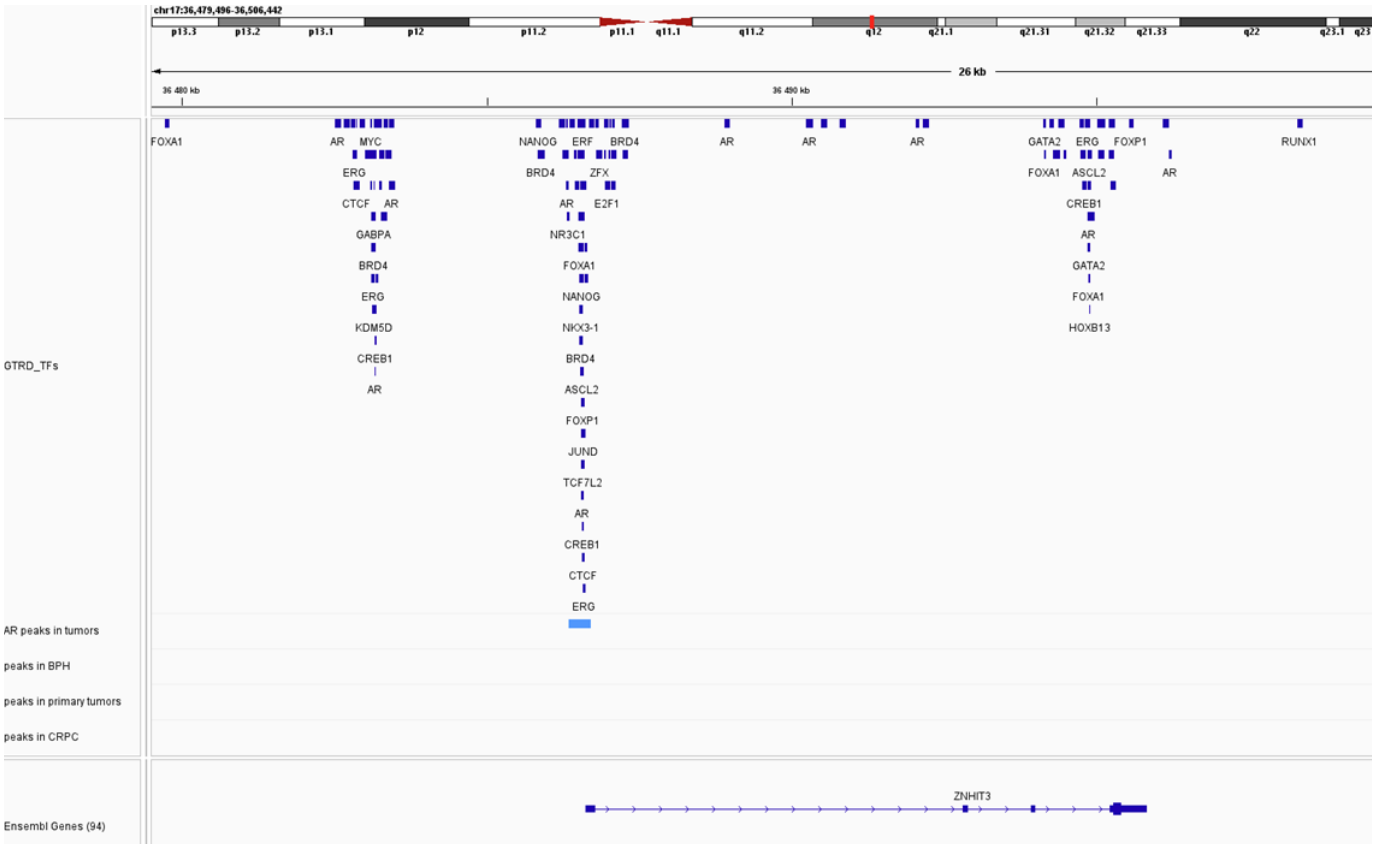

Supplementary Fig. 3. ATAC-seq analysis

The images provide a detailed genomic visualization using IGV (Integrative Genomics Viewer), focusing on TMEM18, FRMPD1, and ZNHIT3. The first panel from the top highlights the binding sites for various transcription factors (TFs) from the GTRD database within this genomic region. The second panel, labeled "AR Peaks in Tumors," shows the presence of AR peaks specifically in tumor samples, marked with blue horizontal bars to indicate regions of significant AR binding. The subsequent panels represent ATAC-seq peaks in BPH, primary tumors, and CRPC samples, displaying the open chromatin regions and indicating accessible chromatin areas in the regulatory region of these genes.

A

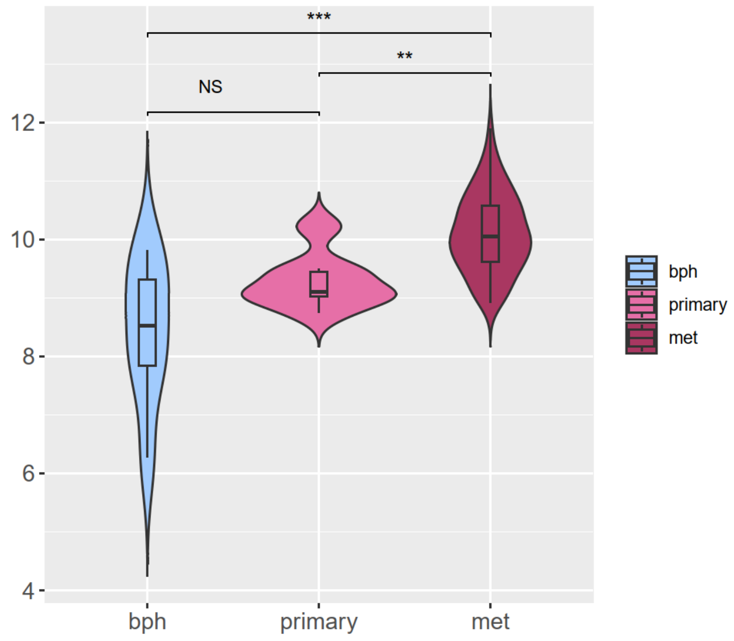

***
**
NS
12
10
8
6
4
bph
primary
met
bph
primary
met

B

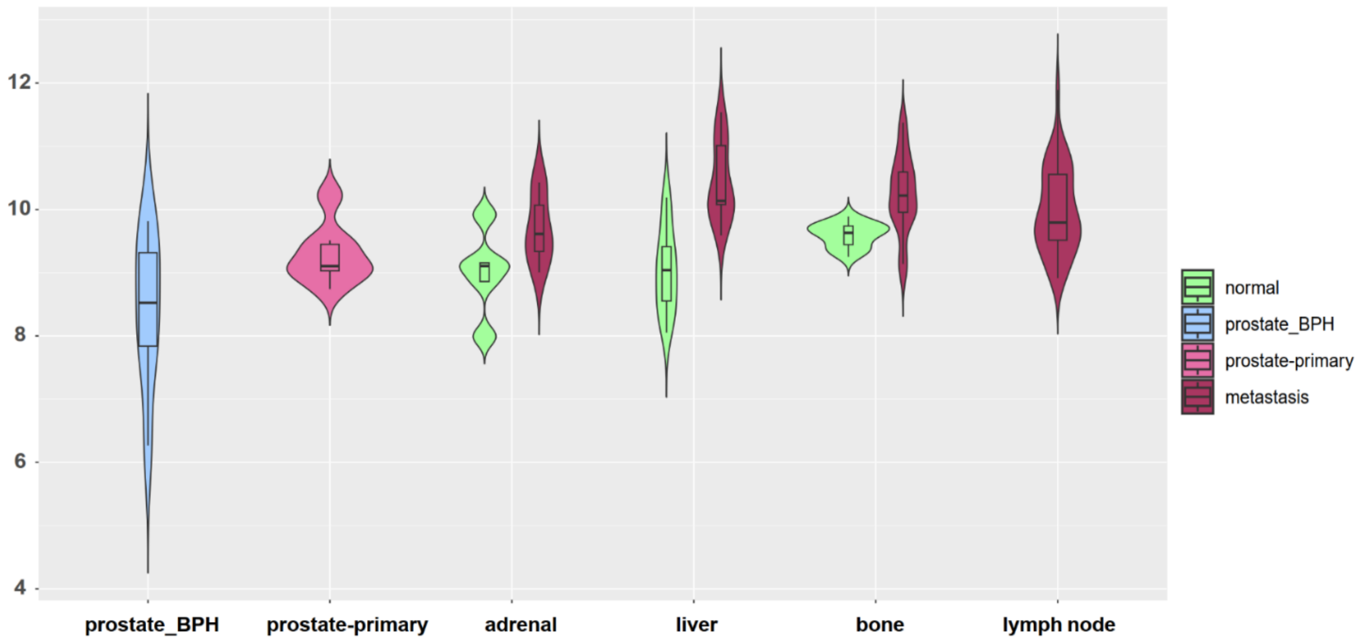

12
10
8
6
4
prostate_BPH
prostate-primary
adrenal
liver
bone
lymph node
normal
prostate_BPH
prostate-primary
metastasis

Supplementary Fig. 4. TMEM18 mRNA expression patterns across tissue types and cancer status. Normalization of read counts was performed using the DESeq2 normalization method. Violin plots are depicted on a log2 (normalized + 1) scale for presentation. Figure 3A, shows TMEM18 expression patterns across different conditions in the cohort. Statistical significance is denoted as follows: NS (not significant), ** ($p < 0.01$) and *** ($p < 0.001$). Figure 3B, shows TMEM18 expression patterns across different tissue types in the mCRPC cohort.

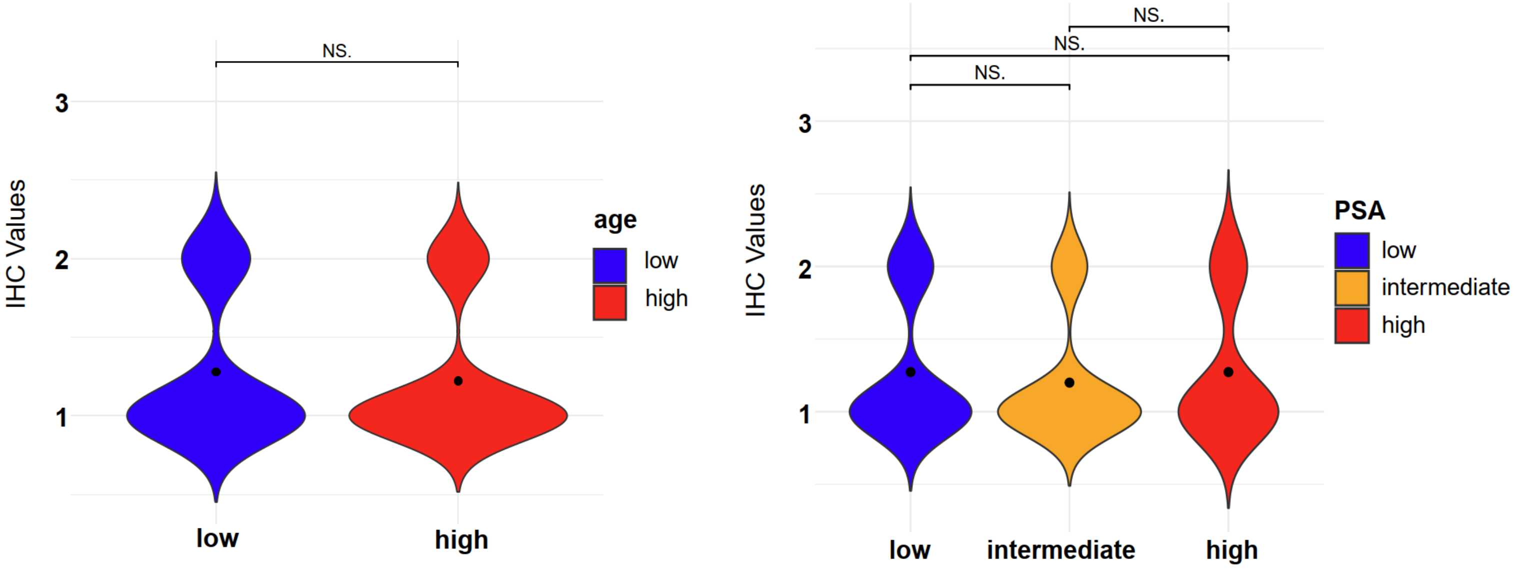

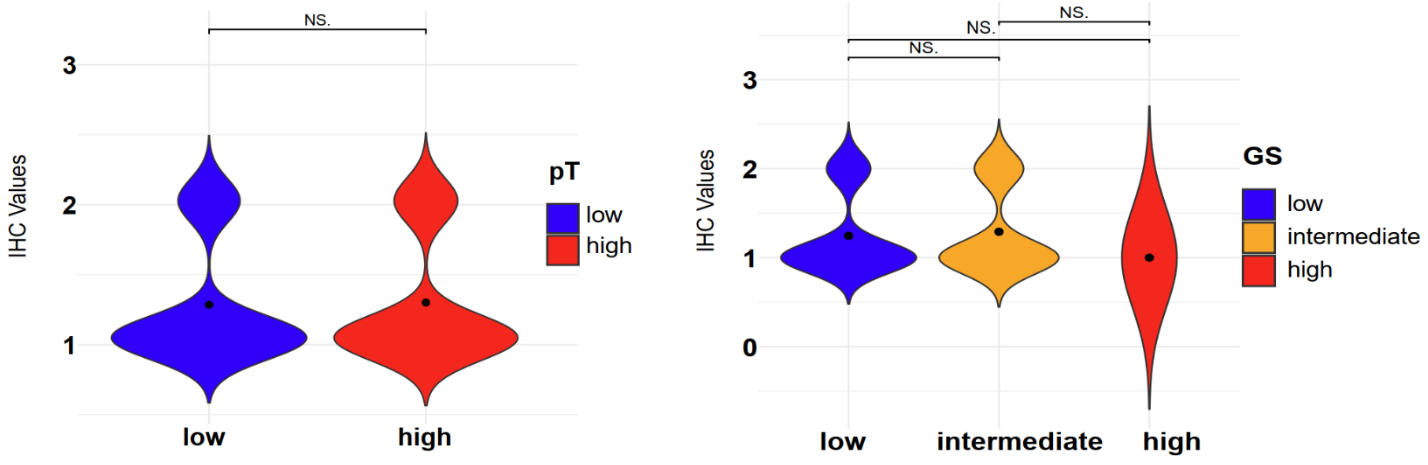

Supplementary Fig. 5. Association of TMEM18 protein expression levels with clinical parameters. TMEM18 expression is presented as IHC intensity values, reflecting protein levels, and is analyzed in relation to various clinical variables, including age (top left), diagnostic PSA levels (top right), Gleason Score (GS; bottom left), and tumor stage (pT; bottom right). Categories for each parameter are stratified (e.g., low, intermediate, high), and statistical significance is indicated. Statistical significance is denoted as follows: NS (not significant) and * ($p < 0.05$). Red dots indicate the mean IHC intensity values for each group.

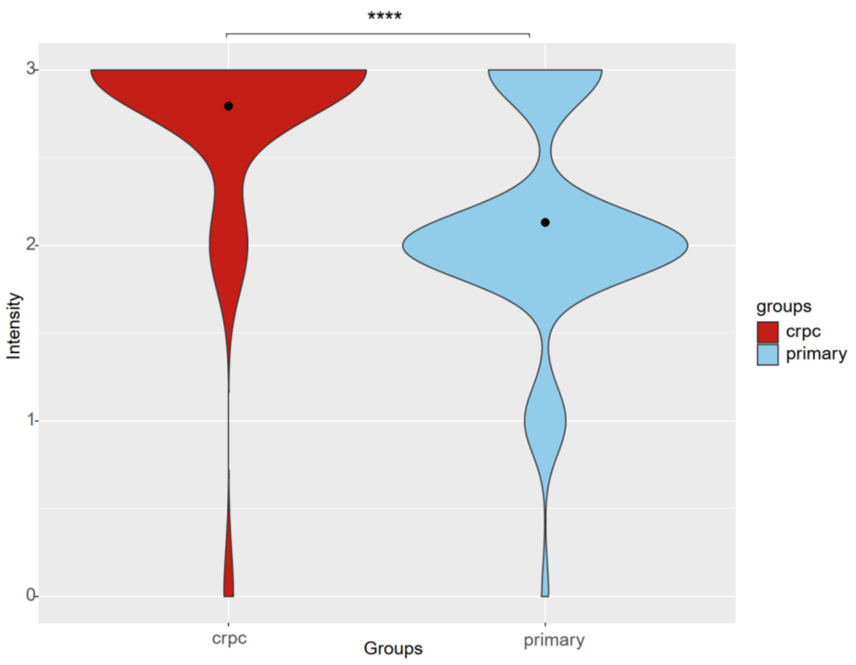

Supplementary Fig .6. Increased TMEM18 protein expression in CRPC compared to primary PCa.

Violin plot showing the intensity of TMEM18 expression in primary PCa (277 samples) and CRPC specimens (34 samples). The black dots represent the median expression levels. The data indicate an increase in TMEM18 expression in CRPC compared to primary PCa. Statistical significance is denoted as follows: **** ($p < 0.0001$).
